# Crystal structure of methyl (*E*)-4-[2-(8-hy­droxy­quinolin-2-yl)vin­yl]benzoate

**DOI:** 10.1107/S205698901601210X

**Published:** 2016-08-05

**Authors:** Yu-Xing Xu, Wei-Ji Hu, Guo-Liang Zhao

**Affiliations:** a College of Chemistry and Life Science, Zhejiang Normal University, Jinhua, Zhejiang 321004, People’s Republic of China; b XingZhi College, Zhejiang Normal University, Jinhua, Zhejiang 321004, People’s Republic of China

**Keywords:** crystal structure, 8-hy­droxy­quinoline derivative, vin­yl, inversion dimer, hydrogen bonding, inversion dimers, C—H⋯π inter­actions

## Abstract

The title compound, C_19_H_15_NO_3_, was synthesized by a Perkin reaction of 2-methyl-8-hy­droxy­quinoline and 4-formyl-2-methyl­benzoate in acetic anhydride under a nitro­gen atmosphere. The mol­ecule has an *E* conformation about the C=C bond, and the quinoline ring system and the benzene ring are inclined to one another by 29.22 (7)°. There is an intra­molecular O—H⋯N hydrogen bond in the 8-hy­droxy­quinoline moiety. In the crystal, mol­ecules are linked by pairs of O—H⋯O hydrogen bonds, forming inversion dimers with an *R*_2_^2^(28) ring motif. The dimers are linked by C—H⋯O hydrogen bonds and C—H⋯π inter­actions, forming sheets parallel to plane (10-1).

## Abstract 2

The title 8-hy­droxy­quinoline derivative has an *E* conformation about the C=C bond, and the quinoline ring system and the benzene ring are inclined to one another by 29.22 (7)°.

## Chemical context   

In recent years, 8-hy­droxy­quinoline and its derivatives have played an important role in coordination chemistry (Albrecht *et al.*, 2008 ▸; Cacciatore *et al.*, 2013 ▸), shown to exhibit biological activity (du Moulinet d’Hardemare *et al.*, 2012 ▸) and have found various applications in the fields of synthetic chemistry (Song *et al.*, 2006 ▸) and organic light-emitting diodes, which have been extensively exploited in the synthesis of luminescent metal complexes (Tang *et al.*, 1987 ▸). It is therefore highly desirable to develop new efficient 8-hy­droxy­quinoline derivatives for use in luminescent metal complexes. In the present work, we report on the synthesis and crystal structure of a new 8-hy­droxy­quinoline derivative, synthesized by the Perkin reaction of 2-methyl-8-hy­droxy­quinoline and 4-formyl-2-methyl­benzoate.
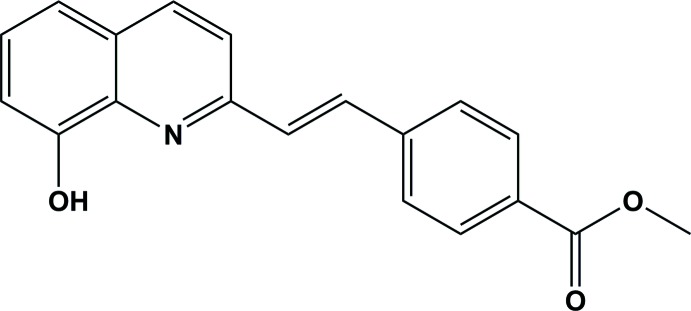


## Structural commentary   

**Figure 1:**
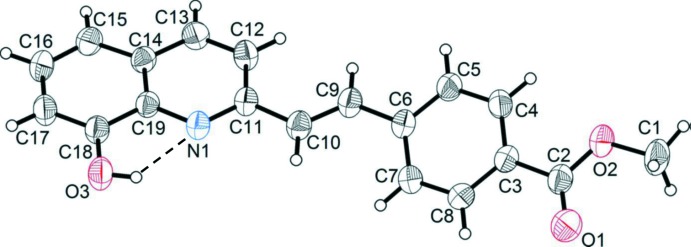
View of the mol­ecular structure of the title compound, showing the atom labelling and 40% probability displacement ellipsoids. The intra­molecular O—H⋯N hydrogen bond is shown as a dashed line (see Table 1 ▸).

The mol­ecular structure of the title compound is shown in Fig. 1 ▸. It contains an 8-hy­droxy­quinoline moiety, with an intra­molecular O—H⋯N hydrogen bond (Fig. 1 ▸ and Table 1 ▸), and a methyl­benzoate unit. They are linked by the C9=C10 bond [1.321 (2) Å] with an *E* conformation. The C11—C10 and C6—C9 bond lengths are 1.463 (2) and 1.466 (2) Å, respectively. These distances are shorter than the standard length of a C—C single bond (*ca* 1.5 Å) because of the conjugate system formed by the C9=C10 bond and the aromatic systems. The quinoline ring system and the benzene ring are inclined to one another by 29.22 (7)°.

## Supra­molecular features   

**Figure 2:**
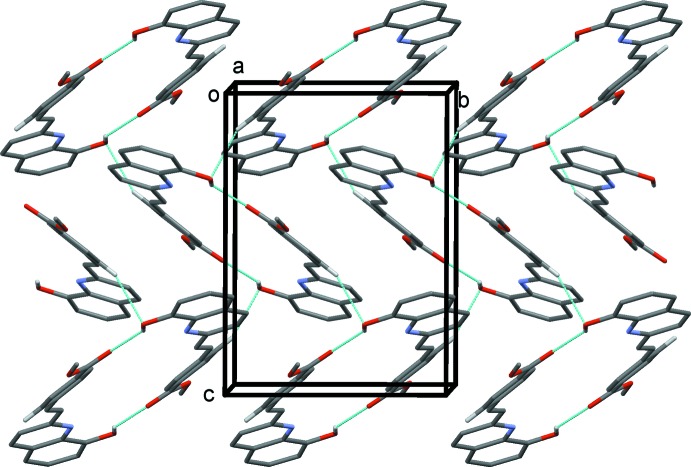
A view along the *a* axis of the 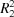
(28) ring motifs in the crystal of the title compound. Hydrogen bonds are shown as dashed lines (see Table 1 ▸), and for clarity only H atoms H3*O* and H5*A* are included.

**Figure 3:**
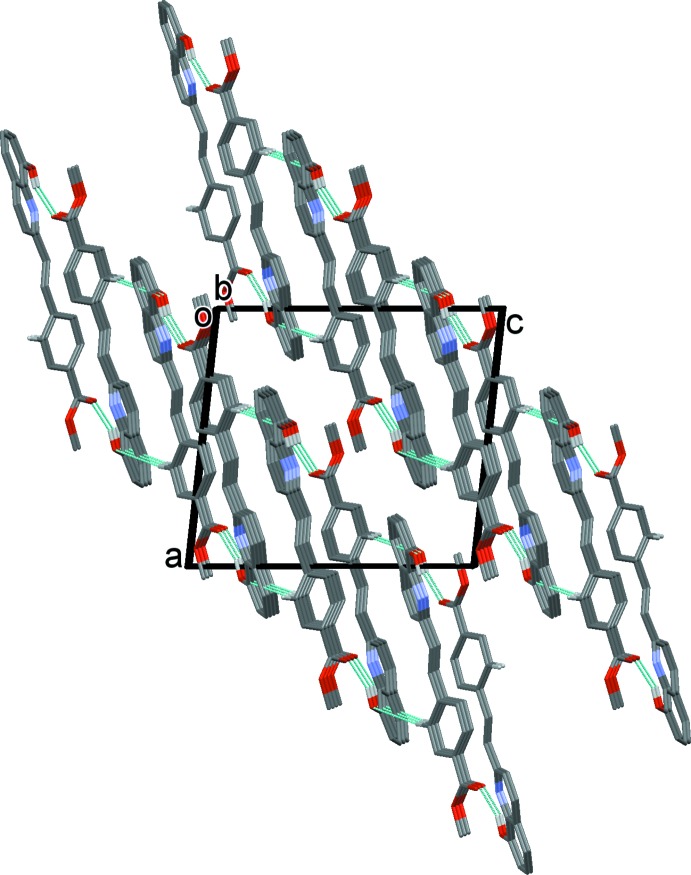
A view along the *b* axis of the crystal packing of the title compound. Hydrogen bonds are shown as dashed lines (see Table 1 ▸) and, for clarity, only H atoms H3*O* and H5*A* are included.

In the crystal, mol­ecules are linked by pairs of O—H⋯O hydrogen bonds, forming inversion dimers with an 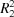
(28) ring motif (Table 1 ▸ and Fig. 2 ▸). The dimers are linked by C—H⋯O hydrogen bonds and C—H⋯π inter­actions, forming sheets parallel to (10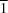); see Table 1 ▸ and Fig. 3 ▸.

## Database survey   

A search of the Cambridge Structural Database (CSD, Version 5.37, last update May 2016; Groom *et al.*, 2016 ▸) for the substructure 2-styrylquinolin-8-ol gave 17 hits; however, certain of these involve bis­(8-hy­droxy­quinolines) or a (9-anthr­yl) moiety. Three compounds are similar to the title compound in the sense that they also have an *E* conformation about the C=C bond, and in the crystal they also form inversion dimers. They include 2-{2-[4-(tri­fluoro­meth­yl)phen­yl]vin­yl}quinolin-8-ol (HUKTOY; Huo *et al.*, 2015 ▸), 2-[2-(4-meth­oxy­phen­yl)vin­yl]quinolin-8-ol (MIMPOP; Yuan *et al.*, 2013 ▸), and 2-[2-(2,4-di­nitro­phen­yl)vin­yl]quinolin-8-ol (WELKEF; Yuan *et al.*, 2013 ▸). In these three compounds, the quinoline and benzene rings are inclined to one another by 36.72 (10) and 16.66 (10)° in HUKTOY (there are two independent mol­ecules in the asymmetric unit), 42.59 (7)° in MIMPOP and 5.63 (6)° in WELKEF, compared to 29.22 (7)° in the title compound.

## Synthesis and crystallization   

The title compound was prepared following reported procedures (Jing *et al.*, 2006 ▸; Yuan *et al.*, 2012 ▸). A mixture of 2-methy-8-hy­droxy­quinoline (1.59 g, 10 mmol), 4-formyl-2-methyl­benzoate (1.64 g, 10 mmol) and acetic anhydride (20 ml) was stirred for 12 h at 423 K under a nitro­gen atmosphere. After cooling it was poured into ice–water (150 ml) and stirred for 1–2 h. Then, the puce solid obtained was filtered and together with tri­ethyl­amine (1 g, 10 mmol) was dissolved in DMF (30 ml) and the mixture stirred for 3 h at 408 K. After cooling, the reaction mixture was concentrated and purified by chromatography on silica gel (petroleum ether/EtOAc = 3/1). The product obtained was dissolved in ethanol, and on slow evaporation of the solvent yellow crystals were obtained within 2 weeks.

## Refinement   

Crystal data, data collection and structure refinement details are summarized in Table 2 ▸. The hy­droxy-H atom was located in a difference Fourier map and freely refined. The C-bound H atoms were positioned geometrically and allowed to ride on their parent atoms: C—H = 0.93–0.96 Å with *U*_iso_(H) = 1.5*U*_eq_(C-meth­yl) and 1.2*U*_eq_(C) for other H atoms.

## Supplementary Material

Crystal structure: contains datablock(s) I, global. DOI: 10.1107/S205698901601210X/su5312sup1.cif

Structure factors: contains datablock(s) I. DOI: 10.1107/S205698901601210X/su5312Isup2.hkl

Supporting information file. DOI: 10.1107/S205698901601210X/su5312Isup3.mol

Supporting information file. DOI: 10.1107/S205698901601210X/su5312Isup4.cml

CCDC reference: 859030

Additional supporting information: crystallographic information; 3D view; checkCIF report

## Figures and Tables

**Table 1:** Hydrogen-bond geometry (Å, °) *Cg*1, *Cg*2 and *Cg*3 are the centroids of rings N1/C11–C14/C19, C3–C8 and C14–C19, respectively.

*D*—H⋯*A*	*D*—H	H⋯*A*	*D*⋯*A*	*D*—H⋯*A*
O3—H3*O*⋯N1	0.86 (2)	2.19 (3)	2.715 (2)	120 (2)
O3—H3*O*⋯O1^i^	0.86 (2)	2.23 (2)	2.901 (2)	136 (2)
C5—H5*A*⋯O3^ii^	0.93	2.57	3.437 (2)	155
C7—H7*A*⋯*Cg*3^iii^	0.93	2.99	3.605 (2)	125
C8—H8*A*⋯*Cg*1^iii^	0.93	2.93	3.559 (2)	126
C15—H15*A*⋯*Cg*2^ii^	0.93	2.83	3.639 (2)	146

Symmetry codes: (i) 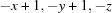; (ii) 
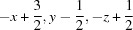
; (iii) 
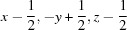
.

**Table 2:** Experimental details

Crystal data	
Chemical formula	C_19_H_15_NO_3_
*M*_r_	305.32
Crystal system, space group	Monoclinic, *P*2_1_/*n*
Temperature (K)	296
*a*, *b*, *c* (Å)	12.0236 (4), 9.7045 (4), 13.2607 (4)
β (°)	96.260 (2)
*V* (Å^3^)	1538.07 (9)
*Z*	4
Radiation type	Mo *K*α
μ (mm^−1^)	0.09
Crystal size (mm)	0.08 × 0.06 × 0.05

Data collection	
Diffractometer	Bruker SMART CCD area-detector
Absorption correction	Multi-scan (*SADABS*; Bruker, 2005 ▸)
*T*_min_, *T*_max_	0.993, 0.996
No. of measured, independent and observed [*I* > 2σ(*I*)] reflections	13068, 3511, 2049
*R*_int_	0.041
(sin θ/λ)_max_ (Å^−1^)	0.651

Refinement	
*R*[*F* ^2^ > 2σ(*F* ^2^)], *wR*(*F* ^2^), *S*	0.046, 0.137, 1.02
No. of reflections	3511
No. of parameters	217
H-atom treatment	H atoms treated by a mixture of independent and constrained refinement
Δρ_max_, Δρ_min_ (e Å^−3^)	0.17, −0.18

Computer programs: *SMART* and *SAINT* (Bruker, 2005 ▸), *SHELXS97*, *SHELXL97* and *SHELXTL* (Sheldrick, 2008 ▸), *Mercury* (Macrae *et al.*, 2008 ▸) and *PLATON* (Spek, 2009 ▸).

## supplementary crystallographic information

### Crystal data

**Table:**

C_19_H_15_NO_3_	*F*(000) = 640
*M**_r_* = 305.32	*D*_x_ = 1.319 Mg m^−^^3^
Monoclinic, *P*2_1_/*n*	Mo *K*α radiation, λ = 0.71073 Å
Hall symbol: -P 2yn	Cell parameters from 1747 reflections
*a* = 12.0236 (4) Å	θ = 2.2–27.6°
*b* = 9.7045 (4) Å	µ = 0.09 mm^−^^1^
*c* = 13.2607 (4) Å	*T* = 296 K
β = 96.260 (2)°	Block, yellow
*V* = 1538.07 (9) Å^3^	0.08 × 0.06 × 0.05 mm
*Z* = 4	

### Data collection

**Table:**

Bruker SMART CCD area-detector diffractometer	3511 independent reflections
Radiation source: fine-focus sealed tube	2049 reflections with *I* > 2σ(*I*)
Graphite monochromator	*R*_int_ = 0.041
phi and ω scans	θ_max_ = 27.6°, θ_min_ = 2.2°
Absorption correction: multi-scan (SADABS; Bruker, 2005)	*h* = −15→14
*T*_min_ = 0.993, *T*_max_ = 0.996	*k* = −12→12
13068 measured reflections	*l* = −17→17

### Refinement

**Table:**

Refinement on *F*^2^	Primary atom site location: structure-invariant direct methods
Least-squares matrix: full	Secondary atom site location: difference Fourier map
*R*[*F*^2^ > 2σ(*F*^2^)] = 0.046	Hydrogen site location: inferred from neighbouring sites
*wR*(*F*^2^) = 0.137	H atoms treated by a mixture of independent and constrained refinement
*S* = 1.02	*w* = 1/[σ^2^(*F*_o_^2^) + (0.0631*P*)^2^ + 0.1091*P*] where *P* = (*F*_o_^2^ + 2*F*_c_^2^)/3
3511 reflections	(Δ/σ)_max_ < 0.001
217 parameters	Δρ_max_ = 0.17 e Å^−^^3^
0 restraints	Δρ_min_ = −0.18 e Å^−^^3^

### Special details

**Table:**

Geometry. All esds (except the esd in the dihedral angle between two l.s. planes) are estimated using the full covariance matrix. The cell esds are taken into account individually in the estimation of esds in distances, angles and torsion angles; correlations between esds in cell parameters are only used when they are defined by crystal symmetry. An approximate (isotropic) treatment of cell esds is used for estimating esds involving l.s. planes.
Refinement. Refinement of F^2^ against ALL reflections. The weighted R-factor wR and goodness of fit S are based on F^2^, conventional R-factors R are based on F, with F set to zero for negative F^2^. The threshold expression of F^2^ > 2sigma(F^2^) is used only for calculating R-factors(gt) etc. and is not relevant to the choice of reflections for refinement. R-factors based on F^2^ are statistically about twice as large as those based on F, and R- factors based on ALL data will be even larger.

### Fractional atomic coordinates and isotropic or equivalent isotropic displacement parameters (Å^2^)

**Table:**

	*x*	*y*	*z*	*U*_iso_*/*U*_eq_	
N1	0.87623 (11)	0.22440 (15)	0.17162 (11)	0.0507 (4)	
O3	1.04463 (12)	0.41136 (15)	0.20030 (12)	0.0678 (4)	
C19	0.97735 (12)	0.17971 (18)	0.21670 (12)	0.0465 (4)	
C3	0.26628 (13)	0.27214 (18)	−0.01100 (13)	0.0496 (4)	
C14	0.99928 (13)	0.04272 (18)	0.24806 (12)	0.0481 (4)	
C11	0.79264 (13)	0.13449 (18)	0.15909 (12)	0.0491 (4)	
O2	0.07181 (10)	0.24711 (14)	−0.02756 (10)	0.0691 (4)	
C6	0.47912 (13)	0.17659 (19)	0.06283 (12)	0.0508 (4)	
C13	0.90819 (14)	−0.04932 (19)	0.23276 (13)	0.0544 (5)	
H13A	0.9178	−0.1412	0.2517	0.065*	
C18	1.06399 (13)	0.27912 (19)	0.23164 (13)	0.0521 (4)	
C12	0.80665 (14)	−0.0046 (2)	0.19061 (13)	0.0549 (5)	
H12A	0.7464	−0.0651	0.1824	0.066*	
C2	0.15483 (14)	0.3263 (2)	−0.05173 (14)	0.0574 (5)	
O1	0.14069 (11)	0.42886 (17)	−0.10263 (13)	0.0939 (5)	
C10	0.68606 (13)	0.18748 (19)	0.11050 (13)	0.0550 (5)	
H10A	0.6873	0.2733	0.0795	0.066*	
C9	0.58827 (13)	0.1246 (2)	0.10668 (13)	0.0550 (5)	
H9A	0.5887	0.0370	0.1352	0.066*	
C8	0.36132 (14)	0.3354 (2)	−0.03918 (13)	0.0587 (5)	
H8A	0.3541	0.4107	−0.0828	0.070*	
C15	1.10686 (14)	0.0073 (2)	0.29359 (13)	0.0581 (5)	
H15A	1.1226	−0.0831	0.3136	0.070*	
C17	1.16678 (14)	0.2423 (2)	0.27826 (15)	0.0611 (5)	
H17A	1.2229	0.3081	0.2901	0.073*	
C7	0.46638 (14)	0.2885 (2)	−0.00345 (14)	0.0587 (5)	
H7A	0.5293	0.3318	−0.0237	0.070*	
C4	0.27801 (14)	0.15980 (19)	0.05325 (14)	0.0564 (5)	
H4A	0.2148	0.1156	0.0721	0.068*	
C16	1.18752 (15)	0.1057 (2)	0.30817 (14)	0.0641 (5)	
H16A	1.2581	0.0816	0.3387	0.077*	
C5	0.38325 (14)	0.11293 (19)	0.08960 (14)	0.0589 (5)	
H5A	0.3900	0.0372	0.1329	0.071*	
C1	−0.04060 (15)	0.2875 (3)	−0.06672 (17)	0.0812 (7)	
H1B	−0.0932	0.2228	−0.0445	0.122*	
H1C	−0.0470	0.2886	−0.1395	0.122*	
H1D	−0.0561	0.3777	−0.0421	0.122*	
H3O	0.9748 (17)	0.414 (3)	0.178 (2)	0.101 (8)	

### Atomic displacement parameters (Å^2^)

**Table:**

	*U*^11^	*U*^22^	*U*^33^	*U*^12^	*U*^13^	*U*^23^
N1	0.0405 (7)	0.0524 (9)	0.0578 (9)	0.0016 (7)	−0.0003 (6)	−0.0028 (7)
O3	0.0509 (8)	0.0541 (8)	0.0935 (10)	−0.0052 (7)	−0.0140 (8)	0.0047 (7)
C19	0.0397 (9)	0.0523 (10)	0.0470 (9)	0.0023 (8)	0.0028 (7)	−0.0005 (8)
C3	0.0447 (9)	0.0557 (11)	0.0471 (9)	0.0034 (8)	−0.0015 (7)	−0.0011 (8)
C14	0.0422 (9)	0.0528 (11)	0.0495 (9)	0.0062 (8)	0.0057 (7)	0.0010 (8)
C11	0.0412 (9)	0.0503 (10)	0.0549 (10)	0.0016 (8)	0.0005 (7)	−0.0059 (8)
O2	0.0414 (7)	0.0864 (10)	0.0774 (9)	0.0056 (7)	−0.0034 (6)	0.0080 (7)
C6	0.0445 (9)	0.0530 (10)	0.0529 (10)	0.0011 (8)	−0.0034 (7)	−0.0051 (8)
C13	0.0527 (10)	0.0471 (10)	0.0631 (11)	0.0035 (8)	0.0056 (8)	0.0009 (8)
C18	0.0430 (9)	0.0531 (11)	0.0590 (10)	0.0007 (8)	0.0007 (8)	0.0001 (9)
C12	0.0457 (9)	0.0515 (11)	0.0663 (11)	−0.0036 (8)	0.0013 (8)	−0.0061 (9)
C2	0.0447 (10)	0.0687 (13)	0.0575 (11)	0.0050 (10)	0.0003 (8)	−0.0010 (10)
O1	0.0597 (9)	0.0984 (12)	0.1208 (13)	0.0137 (8)	−0.0033 (8)	0.0450 (11)
C10	0.0468 (10)	0.0530 (11)	0.0637 (11)	0.0046 (8)	−0.0011 (8)	−0.0015 (9)
C9	0.0446 (9)	0.0549 (11)	0.0635 (11)	0.0026 (8)	−0.0032 (8)	−0.0027 (9)
C8	0.0521 (10)	0.0638 (12)	0.0586 (11)	0.0015 (9)	−0.0004 (8)	0.0133 (9)
C15	0.0456 (10)	0.0620 (12)	0.0661 (11)	0.0115 (9)	0.0034 (8)	0.0087 (9)
C17	0.0424 (10)	0.0680 (13)	0.0713 (12)	−0.0049 (9)	−0.0011 (8)	−0.0012 (10)
C7	0.0427 (9)	0.0703 (13)	0.0624 (11)	−0.0039 (9)	0.0027 (8)	0.0070 (10)
C4	0.0436 (9)	0.0584 (11)	0.0657 (11)	−0.0046 (8)	−0.0005 (8)	0.0035 (9)
C16	0.0404 (9)	0.0767 (15)	0.0731 (12)	0.0064 (9)	−0.0044 (8)	0.0075 (11)
C5	0.0495 (10)	0.0554 (11)	0.0691 (12)	−0.0004 (8)	−0.0054 (8)	0.0110 (9)
C1	0.0402 (10)	0.1128 (19)	0.0881 (15)	0.0112 (11)	−0.0039 (9)	0.0050 (13)
H3O	0.039 (13)	0.12 (2)	0.14 (2)	−0.025 (14)	−0.010 (13)	0.009 (16)

### Geometric parameters (Å, º)

**Table:**

N1—C11	1.328 (2)	C18—C17	1.368 (2)
N1—C19	1.3657 (19)	C12—H12A	0.9300
O3—C18	1.361 (2)	C2—O1	1.204 (2)
O3—H3O	0.86 (2)	C10—C9	1.321 (2)
C19—C14	1.409 (2)	C10—H10A	0.9300
C19—C18	1.418 (2)	C9—H9A	0.9300
C3—C4	1.381 (2)	C8—C7	1.377 (2)
C3—C8	1.384 (2)	C8—H8A	0.9300
C3—C2	1.485 (2)	C15—C16	1.360 (3)
C14—C15	1.409 (2)	C15—H15A	0.9300
C14—C13	1.411 (2)	C17—C16	1.398 (3)
C11—C12	1.417 (3)	C17—H17A	0.9300
C11—C10	1.463 (2)	C7—H7A	0.9300
O2—C2	1.326 (2)	C4—C5	1.381 (2)
O2—C1	1.448 (2)	C4—H4A	0.9300
C6—C5	1.388 (2)	C16—H16A	0.9300
C6—C7	1.395 (3)	C5—H5A	0.9300
C6—C9	1.466 (2)	C1—H1B	0.9600
C13—C12	1.357 (2)	C1—H1C	0.9600
C13—H13A	0.9300	C1—H1D	0.9600
			
C11—N1—C19	118.15 (15)	C11—C10—H10A	117.0
C18—O3—H3O	105.2 (17)	C10—C9—C6	127.66 (18)
N1—C19—C14	123.90 (15)	C10—C9—H9A	116.2
N1—C19—C18	116.78 (16)	C6—C9—H9A	116.2
C14—C19—C18	119.32 (14)	C7—C8—C3	120.90 (17)
C4—C3—C8	119.02 (15)	C7—C8—H8A	119.5
C4—C3—C2	122.07 (17)	C3—C8—H8A	119.5
C8—C3—C2	118.91 (17)	C16—C15—C14	119.79 (18)
C15—C14—C19	119.34 (16)	C16—C15—H15A	120.1
C15—C14—C13	124.70 (17)	C14—C15—H15A	120.1
C19—C14—C13	115.94 (14)	C18—C17—C16	120.02 (17)
N1—C11—C12	121.65 (14)	C18—C17—H17A	120.0
N1—C11—C10	116.03 (16)	C16—C17—H17A	120.0
C12—C11—C10	122.32 (15)	C8—C7—C6	120.51 (17)
C2—O2—C1	117.01 (16)	C8—C7—H7A	119.7
C5—C6—C7	118.07 (15)	C6—C7—H7A	119.7
C5—C6—C9	118.56 (17)	C5—C4—C3	120.21 (17)
C7—C6—C9	123.36 (17)	C5—C4—H4A	119.9
C12—C13—C14	120.40 (17)	C3—C4—H4A	119.9
C12—C13—H13A	119.8	C15—C16—C17	121.54 (16)
C14—C13—H13A	119.8	C15—C16—H16A	119.2
O3—C18—C17	120.02 (17)	C17—C16—H16A	119.2
O3—C18—C19	120.01 (14)	C4—C5—C6	121.28 (17)
C17—C18—C19	119.96 (17)	C4—C5—H5A	119.4
C13—C12—C11	119.92 (16)	C6—C5—H5A	119.4
C13—C12—H12A	120.0	O2—C1—H1B	109.5
C11—C12—H12A	120.0	O2—C1—H1C	109.5
O1—C2—O2	123.39 (16)	H1B—C1—H1C	109.5
O1—C2—C3	124.26 (18)	O2—C1—H1D	109.5
O2—C2—C3	112.33 (17)	H1B—C1—H1D	109.5
C9—C10—C11	126.00 (18)	H1C—C1—H1D	109.5
C9—C10—H10A	117.0		
			
C11—N1—C19—C14	2.0 (2)	C8—C3—C2—O2	173.08 (16)
C11—N1—C19—C18	−178.23 (15)	N1—C11—C10—C9	−167.28 (17)
N1—C19—C14—C15	179.78 (15)	C12—C11—C10—C9	13.1 (3)
C18—C19—C14—C15	0.0 (2)	C11—C10—C9—C6	177.07 (16)
N1—C19—C14—C13	−1.6 (2)	C5—C6—C9—C10	−162.92 (18)
C18—C19—C14—C13	178.58 (15)	C7—C6—C9—C10	16.0 (3)
C19—N1—C11—C12	−0.4 (2)	C4—C3—C8—C7	−0.5 (3)
C19—N1—C11—C10	179.96 (14)	C2—C3—C8—C7	−179.86 (16)
C15—C14—C13—C12	178.19 (17)	C19—C14—C15—C16	1.0 (3)
C19—C14—C13—C12	−0.3 (2)	C13—C14—C15—C16	−177.45 (18)
N1—C19—C18—O3	−0.9 (2)	O3—C18—C17—C16	−178.39 (18)
C14—C19—C18—O3	178.95 (16)	C19—C18—C17—C16	2.1 (3)
N1—C19—C18—C17	178.64 (16)	C3—C8—C7—C6	−0.6 (3)
C14—C19—C18—C17	−1.6 (2)	C5—C6—C7—C8	1.3 (3)
C14—C13—C12—C11	1.8 (3)	C9—C6—C7—C8	−177.65 (17)
N1—C11—C12—C13	−1.5 (3)	C8—C3—C4—C5	0.8 (3)
C10—C11—C12—C13	178.16 (16)	C2—C3—C4—C5	−179.85 (16)
C1—O2—C2—O1	0.9 (3)	C14—C15—C16—C17	−0.5 (3)
C1—O2—C2—C3	−177.71 (15)	C18—C17—C16—C15	−1.1 (3)
C4—C3—C2—O1	175.11 (19)	C3—C4—C5—C6	−0.1 (3)
C8—C3—C2—O1	−5.6 (3)	C7—C6—C5—C4	−1.0 (3)
C4—C3—C2—O2	−6.3 (3)	C9—C6—C5—C4	178.01 (16)

### Hydrogen-bond geometry (Å, º)

*Cg*1, *Cg*2 and *Cg*3 are the centroids of rings N1/C11–C14/C19, C3–C8 and C14–C19, respectively.

**Table:**

*D*—H···*A*	*D*—H	H···*A*	*D*···*A*	*D*—H···*A*
O3—H3*O*···N1	0.86 (2)	2.19 (3)	2.715 (2)	120 (2)
O3—H3*O*···O1^i^	0.86 (2)	2.23 (2)	2.901 (2)	136 (2)
C5—H5*A*···O3^ii^	0.93	2.57	3.437 (2)	155
C7—H7*A*···*Cg*3^iii^	0.93	2.99	3.605 (2)	125
C8—H8*A*···*Cg*1^iii^	0.93	2.93	3.559 (2)	126
C15—H15*A*···*Cg*2^ii^	0.93	2.83	3.639 (2)	146

Symmetry codes: (i) −*x*+1, −*y*+1, −*z*; (ii) −*x*+3/2, *y*−1/2, −*z*+1/2; (iii) *x*−1/2, −*y*+1/2, *z*−1/2.
